# Data Loss Reconstruction Method for a Bridge Weigh-in-Motion System Using Generative Adversarial Networks

**DOI:** 10.3390/s22030858

**Published:** 2022-01-23

**Authors:** Yizhou Zhuang, Jiacheng Qin, Bin Chen, Chuanzhi Dong, Chenbo Xue, Said M. Easa

**Affiliations:** 1 College of Civil Engineering, Zhejiang University of Technology, Hangzhou 310014, China; yizhouzhuang@zjut.edu.cn (Y.Z.); jiachengqin@zjut.edu.cn (J.Q.); xuechenbo@aliyun.com (C.X.); 2 Department of Civil Engineering, Zhejiang University City College, Hangzhou 310015, China; chenbin@zucc.edu.cn; 3 Yangtze Delta Institute of Urban Infrastructure, Hangzhou 310005, China; 4 Department of Civil, Environmental, and Construction Engineering, University of Central Florida, Orlando, FL 32816, USA; 5 Department of Civil Engineering, Ryerson University, Toronto, ON M5B 2K3, Canada; seasa@ryerson.ca

**Keywords:** bridge weigh-in-motion system, data loss, data reconstruction, generative adversarial network, convolutional neural network, deep learning

## Abstract

In the application of a bridge weigh-in-motion (WIM) system, the collected data may be temporarily or permanently lost due to sensor failure or system transmission failure. The high data loss rate weakens the distribution characteristics of the collected data and the ability of the monitoring system to conduct assessments on bridge condition. A deep learning-based model, or generative adversarial network (GAN), is proposed to reconstruct the missing data in the bridge WIM systems. The proposed GAN in this study can model the collected dataset and predict the missing data. Firstly, the data from stable measurements before the data loss are provided, and then the generator is trained to extract the retained features from the dataset and the data lost in the process are collected by using only the responses of the remaining functional sensors. The discriminator feeds back the recognition results to the generator in order to improve its reconstruction accuracy. In the model training, two loss functions, generation loss and confrontation loss, are used, and the general outline and potential distribution characteristics of the signal are well processed by the model. Finally, by applying the engineering data of the Hangzhou Jiangdong Bridge to the GAN model, this paper verifies the effectiveness of the proposed method. The results show that the final reconstructed dataset is in good agreement with the actual dataset in terms of total vehicle weight and axle weight. Furthermore, the approximate contour and potential distribution characteristics of the original dataset are reproduced. It is suggested that the proposed method can be used in real-life applications. This research can provide a promising method for the data reconstruction of bridge monitoring systems.

## 1. Introduction

### 1.1. Background

In recent years, weigh-in-motion (WIM) systems have been increasingly used in bridge structural health monitoring (SHM) [1,2]. There are two main types of WIM that are applied to bridges: pavement WIM systems and bridge weigh-in-motion (B-WIM) systems [3]. The pavement WIM system embeds sensors in the pavements of bridges and detects the axle and vehicle weights by measuring the generated responses when the vehicle passes through the pavement with the embedded sensors. The system consists of a set of sensors and electronic instruments containing software. B-WIM uses the entire bridge as a scale to measure the weights of vehicles, inferring the axle and vehicle weights by measuring the responses of certain points on the bridge. As B-WIM requires a system that is tailored to the specific conditions of the bridge [4,5,6], it is difficult for it to be widely implemented on a large proportion of bridges. The traditional pavement WIM system has been widely implemented in bridge WIM practices. Bridge WIM systems measure the emergence time of a moving vehicle and its dynamic tire forces. They can obtain vehicle weight, axle weight, speed, arrival time, lane, and other vehicle parameters, such as vehicle type and display and storage, without affecting the regular operation of the vehicle or displaying and storing them. Bridge WIM systems can accurately measure the static parameters (e.g., gross vehicle weight, axle weight, and vehicle type) and monitor the dynamic parameters (e.g., vehicle arrival time, travel speed, acceleration, and lane information). The bridge WIM can directly identify overloaded vehicles and collect data for traffic planning and economic surveys [7]. The data obtained from this system consist of two main components: a variation characteristic of the bridge, usually strain, and axle data [8]. To perform data conversion calculations, the conventional commercial systems measure the response induced by the vehicle passing through sensors that are attached under the structure to obtain axle data, etc. The information provided by these components (i.e., measured strain) is then converted into axle load by applying an algorithm. Jia et al. proposed the application of a neural network that identifies the ideal sample that is obtained from the load sensor closest to the tire–road contact area [9]. More recently, moving force identification (MFI) technology has been applied to measure signals and improve the accuracy of axle weight measurements [10]. Chatterjee et al. used the concept of continuous wavelet transform to accurately identify the axles of moving vehicles in the time domain [11]. Zhao et al. improved the accuracy of bridge WIM by filtering the strain signals corresponding to dynamic load, boundary conditions, and the vibration of trucks [12].

During the operation of the bridge WIM system, measured data may be temporarily or permanently lost due to sensor failure or transmission failure. The loss of measurement data can have serious consequences. Fan et al. pointed out that the impact of a loss of 0.5–2.5% of the dataset on subsequent data analysis is similar to that brought about by 10% observation noise [13]. Pei et al. [14] measured the extent and amount of data loss for different wireless sensors operating in unlicensed industrial, scientific, and medical (ISM) bands in various structural monitoring applications. Kurata et al. [15] identified data loss in building risk monitoring that uses wireless sensor networks. Meyer et al. [16] also assessed data loss associated with using wireless sensors to measure temperature data over several periods. For example, in excitation tests using smart wireless sensors mounted on steel bridges in Austria, data losses were up to 40% [17]. Linderman et al. when referring to the Imote2 platform, stated that radio interference was the main reason for wireless smart sensor data loss [18]. Further examples of interference include other devices running on the same frequency, weather issues such as rain and lightning, poor installation and antenna orientation, long transmission distance, and hardware issues. Nagayama et al. studied these effects through simulations and experiments. Their results showed that 0.5% data loss and 5% noise addition have similar effects on power spectral density (PSD) estimates [19,20].

Replacing faulty sensors is currently a standard method of solving this problem; however, finding and resolving sensor failures is labor-intensive and time-consuming. In addition, the embedded sensors cannot be replaced. Therefore, finding a method for completing or reconstructing a dataset in the case of missing data has become one of the current needs.

Grakovski and Pilipovec et al. reconstructed the weight data of each wheel in a vehicle dynamic weighing measurement by a fiber optic sensor (FOS), based on the function of the change of the optical signal parameters caused by the deformation of the fiber under the action of the weight of the traversing vehicle [21]. This sensor is compact and sensitive but requires special optical fibers and is also costly; therefore, it is not widely used. The missing data complementation methods that are often used at this stage include the time-regularized matrix decomposition and spectral regularization algorithms. Yu et al. proposed the time regularized matrix decomposition (TRMF) framework to solve the time series forecasting problem for modern applications, such as climatology and demand forecasting [22]. This method is well suited for high-dimensional time series data that has many missing values. Stekhoven et al. proposed and evaluated an iterative interpolation method based on random forests (MissForest) [23]. They used the method in the biological domain and MissForest could successfully handle missing values when the artificially introduced missing values were between 10% and 30%. Mazumder et al. proposed a simple and efficient algorithm to minimize the reconstruction error with bounded kernel parametrization [24]. The algorithm iteratively replaces the missing elements with elements obtained from the soft threshold singular value decomposition (SVD) to provide a series of regularized low-rank solutions for large-scale matrix completion problems. Hapfelmeier et al. compared random forest algorithms and multiple interpolation methods within the context of missing database problems in the field of liver surgery [25]. It was found that random forests are usually suitable for data from datasets that had not been preprocessed. In contrast, multiple interpolation methods performed better in complementing preprocessed datasets.

With the development of deep learning in recent years, new approaches to solving the above problems have emerged. Among all deep learning frameworks, convolutional neural networks (CNNs) can adaptively learn high-level features and potential distribution patterns of data by scanning local regions of many data matrices with convolutional kernels [26]. Conventional data reconstruction using CNNs generally uses an encoder–decoder structure, i.e., convolution–deconvolution. The encoder uses a convolution kernel to perform several compressions and feature extractions on a vast dataset. This results in a smaller multichannel matrix, which is then reconstructed using a decoder as the input. This feature is well suited for time series data compression and reconstruction. Li et al. proposed a deep learning-based framework for the estimation of multimodal imaging data [27]. The framework takes the form of a convolutional neural network and when trained on subjects with all modalities, the network can estimate the output modality provided by the input modality (where the input and output modalities are magnetic resonance imaging (MRI) and positron emission tomography (PET) images, respectively). Zahavy et al. constructed a supervised neural network with convolutional and fully connected layers and used a deep neural network to reconstruct ultrashort pulses that were capable of diagnosing very faint pulses [28]. Cong et al. proposed a deep learning-based computed tomography (CT) image reconstruction method that solves the mismatch between computational and physical models [29]. The method utilizes a nonlinear transformation from big data to projection data in order to precisely match the linear integral model to achieve monochromatic imaging, effectively overcoming the beam hardening problem in this field. Zhang et al. utilized two fully connected neural networks that were trained using simulated data and Gaussian noise [30]. The method used MR fingerprinting arterial spin labeling (MRF-ASL) data from healthy subjects and patients with moyamoya disease. The results demonstrated that reconstructing MRF-ASL with fully connected neural networks is faster and more reproducible than traditional DM methods. Glisic and Kim et al. proposed a structural response recovery method that was based on convolutional neural networks [31]. The method aimed to recover missing structural strain response communication errors when the structural response was not collected due to sensor failure, data loss or other reasons. Fan et al. used an autoencoder to reconstruct incomplete vibration measurement data that had a high loss rate [32]. The time series structural acceleration data in raw form were set as the input and the output of the autoencoder. According to their study, modal identification achieved good agreement between the original and reconstructed data. Ni et al. proposed a new method for data compression and reconstruction using autoencoders [33]. The multisource data obtained from actual measurements could be stored with very low compression ratios and restored to the standard data with high accuracy in the time and frequency domains. Wang and Cha proposed an unsupervised deep learning method to detect impairment using autoencoders by reconstructing the data and comparing it with the impaired response [34]. This unsupervised deep learning approach provided a stable and robust damage detection performance using acceleration measurements that were collected from the intact structure.

A generative adversarial network (GAN) is an unsupervised learning technique that uses two competing neural networks (a generator and a discriminator) to generate new datasets. The generator generates the data while the discriminator discriminates them for veracity. Chandak et al. used a generative adversarial network structure for image complementation, generating coarse blocks to fill in missing regions in distorted images [35]. The network also used the residual learning process to refine the generated images further, thus providing better complete images for computer vision applications. Xiong and Chen used a GAN to generate all types of human evoked loads, including walking, jumping, and bouncing loads [36]. In this method, the model was trained using experimentally collected human loads. The trained generator could reconstruct high-quality signals in both the time and frequency domains that were similar to the actual data. Kim et al. proposed the use of a GAN to reconstruct multiple lost structural signals [37]. The model was first trained with actual signals to determine the optimal convolutional kernel weights, laying the foundation for the reconstruction of the lost data. The reconstructed lost signals were within the acceptable range of reconstruction error. Lei and Sun used a GAN to reconstruct the lost data of a structural health monitoring system [38]. The reconstruction of the faulty sensor data was investigated in both the time and frequency domains using the responses of the remaining functional sensors. The results showed that the final reconstructed signal matched the actual signal in both the time and frequency domains. A considerable number of implied features and distribution patterns exist in the measured time series dataset. With the help of generators and discriminators, a GAN can correctly reconstruct the missing parts of the time signal. The generator is responsible for capturing the overall structure of the signal and the distribution patterns. On the other hand, the discriminator tries to optimize the generator by discriminating the generated data to make the reconstruction look realistic.

### 1.2. Objective and Scope

However, most of the applications and experiments in past studies have been carried out in ideal measurement environments to ensure adequate accuracy. In addition, when the validation experiments of these new methods have been carried out, the measurement time span was generally short, thereby avoiding most of the disadvantages that affect the measurement accuracy and stability. However, when conducting experiments to deploy a real-world system, adverse contingencies may affect the quality of the measurements. Therefore, it is very necessary to reconstruct the dataset over a long time span.

This study proposes a novel deep convolutional GAN to reconstruct the lost vehicle weight data in a bridge WIM system. The proposed generative adversarial network methodology is described in detail in Section 2. In that section, we describe the overall GAN-based architecture, including the selected layer combinations and activation functions that were used in the network. The generative and discriminative loss functions that were used to accurately reconstruct the lost dataset are also presented. In Section 3, the field applicability of the proposed GAN model is verified using the vehicle weight–time data that were measured by the bridge WIM system of the Hangzhou Jiangdong Bridge. Finally, appropriate conclusions are drawn in the “Conclusion” section. This research can provide a promising method for the data reconstruction of bridge monitoring systems.

## 2. Proposed Data Loss Reconstruction Method Using a GAN

### 2.1. Architecture

**Figure 1:**
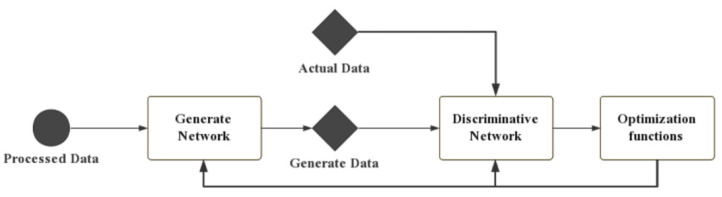
The general form of a GAN.

A generative adversarial network comprises two parts: a generator (G) and a discriminator (D). A new dataset that is similar to the actual dataset is generated by processing several convolution layers and several layers of deconvolution in G. D is just a classifier; it takes actual or generated data as input and tries to distinguish them as true or false. Then, the classification results are transformed into reconstruction loss and antagonistic loss to update G and D. The task of D is to distinguish the samples that were generated by G from the training data. Moreover, G is confounded by generating samples whose distribution is close to the training data distribution. The operation flow of a GAN is shown in Figure 1. In this paper, both the generator and discriminator were constructed using a CNN. In the present study, the inputs for G and D were vehicle weight datasets that were composed of collected time series data, i.e., the vehicle weight data and axle load data of all vehicles crossing the bridge within a certain period.

#### 2.1.1. Generated Network

The generated network is constructed using a basic framework of convolution–deconvolution. The first half is a convolutional neural network comprising several layers and the second half is a deconvolutional neural network comprising several layers. The convolutional layers have the role of extracting features in-depth at different scales [39].

In order to improve the training efficiency, the method of training the network with small batches, rather than large datasets, was used. Therefore, a batch normalization (BN) layer was added after the convolutional layer in order to improve the stability of the training [40]. In addition, an activation function needs to be introduced after each convolutional layer in order to cope with the needs of nonlinear problems. In this paper, ReLU and Leaky ReLU were selected as the activation functions of the generator [41]. After the last convolution operation in the convolution part, a multichannel matrix of size 11 is generated. The multichannel matrix contains the potential features of the existing data and the distribution pattern between the missing data and the existing data.

The second half of the generator is divided into several layers of deconvolution layers, whose function is to expand the multichannel matrix that is obtained by convolution into a single-channel dataset matrix. The structure is similar to that of the convolutional layers, where each deconvolutional layer is followed by BN and activation function layers.

#### 2.1.2. Discriminative Network

In this paper, the discriminative network consisted of several convolutional layers and a BN layer with an activation function. First, the discriminator received the actual or reconstructed dataset (generated by the generator). Then, a multichannel matrix of 1 × 1 was obtained through the convolution step of the discriminator. Note that in the last layer of the discriminator, the activation function was a sigmoid function instead of the ReLU or Leaky ReLU function. This was useful because the sigmoid function could map an actual number to the range (0, 1) and could be used to perform the binary classification task, thus satisfying the needs of the discriminator as a classifier.

### 2.2. Loss Function

When reconstructing lost data, it is necessary to examine both the underlying features and the data distribution patterns. The total training loss that is obtained from the discriminator is very complex and it is not easy to update the whole network. Therefore, the method of dividing the total loss into generator loss and discriminator loss is usually used [42]. A good model should weigh both losses to make them optimal at the same time.

#### 2.2.1. Generator Loss

The generator loss mainly captures the instinctive structure of the existing dataset and the missing part of the dataset and its correlation. At this stage, L1 and L2 paradigms are often used to measure the distance between actual and false data [43]. Considering that the L2 parametric can prevent overfitting and improve the generalization ability of the model to obtain better training efficiency and stability, the normalized L2 parametric was used as the reconstruction loss function in this paper, as shown in the following equation:

(1) $$\;L_{rec}\left(x\right)=\;||\hat{M}\;⊙{\left(\chi -G\left(\left(1-\hat{M}\right)⊙\chi \right)\right)||}^{2}$$

where χ denotes the actual dataset; G denotes the output data of the generator; $\hat{M}$ denotes the binary mask corresponding to the missing part of the data, which has a value of 1 when the data are missing and 0 for the existing data; ⊙ denotes the pixel-level multiplication; and ∥ ∥ denotes the Euclidean parametrization.

#### 2.2.2. Discriminator Loss

The discriminator loss is a vital part of the training process, which updates the discriminator to instruct the generator to add latent features to the fuzzy rough outline. The discriminator tries to detect the maximum difference between the real data and the reconstructed data, while the generator tries to create more realistic reconstructed data to minimize the difference between them. By combining the two loss functions, the optimization becomes as follows:

(2) $$\underset{G}{\min}\;\underset{D}{\max}\;E\left[\log\left(D\left(x\right)\right)\right]+E\left[\log\left(1-D\left(G\left(x\right)\right)\right)\right]$$

In practice, the stochastic gradient descent algorithm is used to alternately optimize the generator and the discriminator [44]. The adversarial loss can be expressed as:

(3) $$\;L_{adv}=\underset{D}{\max}\;E\left[\log\left(D\left(x\right)\right)+\log\left(1-D\left(G\left(\left(1-\;\hat{M}\;\right)⊙x\right)\right)\right)\right]$$

### 2.3. Precision Measurement Parameters

The reconstruction error is expressed in the form of the root mean square error (RMSE) and the coefficient of determination ($R^{2}$). RMSE is the standard deviation of the residuals, which is a measure of the distance between the reconstructed data and the real data. $R^{2}$ is a statistical measure that is used to analyze the similarity between two signals and ranges from 0 to 1. A good model should have a low RSME value and a high $R^{2}$ value. The RSME and $R^{2}$ are given by:

(4) $$\mathrm{RMSE}=\sqrt{\frac{1}{m}\overset{m}{\underset{i=1}{\sum }}{\left(y_{i}-\hat{y_{l}}\right)}^{2}}$$

(5) $${\;R}^{2}=1-\frac{\sum_{i=1}^{m}{\left(y_{i}-\hat{y_{l}}\right)}^{2}}{\sum_{i=1}^{m}{\left(y_{i}-\;\bar{y}\;\right)}^{2}}$$

where m denotes the number of data; $y_{i}$ denotes the data in the real dataset; $\hat{y_{l}}$ denotes the data in the reconstructed dataset; and $\bar{y_{l}}$ denotes the average value of the real dataset.

## 3. Experimental Verification

### 3.1. Engineering Background

This paper took the Hangzhou Jiangdong Bridge as the research object and selected the data that were collected by the automatic weighing system of this bridge from June to December 2020 for analysis. The Jiangdong Bridge (Qianjiang Nine Bridges) is located in the northeastern corner of Hangzhou city, starting from Xiasha in the west and connecting to Xiaoshan in the east. The bridge is 4.33 km long, of which the bridge range is 3552.66 m, the bridge across the river is 2253 m, and the approach bridge on both sides is 1299.66 m. The main road is a two-way 8-lane road with a 2-m wide sidewalk on each side. The design speed is *v* = 80 km/h, which is normal for an urban expressway. The main bridge structure consists of two self-anchored suspension bridges, a prestressed concrete rigid bridge, and a non-navigable hole in the river for the prestressed concrete continuous beam bridge. The WIM uses a BGK-4000 strain gauge, installed at 80 m and 27 m away from the main bridge span. After the measured strain data are converted to stress, the vehicle weight and axle load data are obtained. The SHM system of the Jiangdong Bridge focuses on the deflection system of the main bridge, the static alignment monitoring, and the deflection of the main tower, and the stress of the key parts is monitored comprehensively.

### 3.2. Statistical Overview

**Figure 2:**
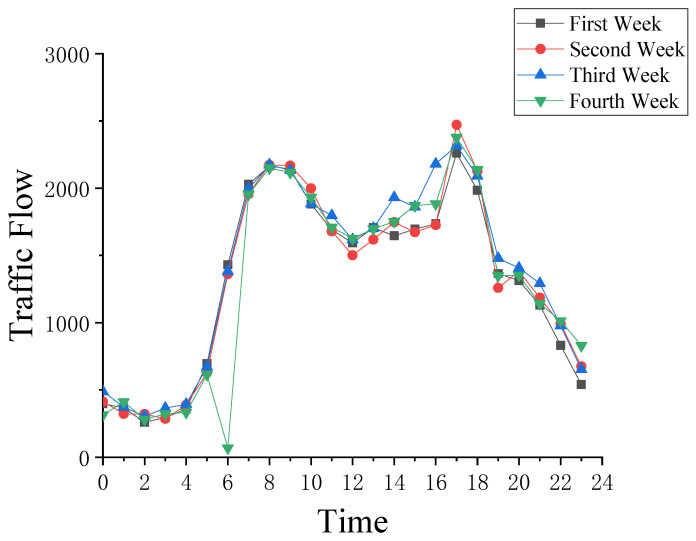
The Jiangdong Bridge traffic flow in August 2020.

The collected data from the bridge WIM system were organized by month and processed using the method described in the previous section. The data for each Tuesday in August 2020 were analyzed in detail and the results are shown in Figure 2.

From the graph, the data from the first week to the third week of August 2020 are 1360 to 1450. However, at 6 a.m. on the Tuesday of the fourth week, the traffic flow is only 12 and these data do not match the actual situation. This phenomenon indicates that the traffic flow data collected at 6:00 a.m. on the Tuesday of the fourth week contained missing data. In this paper, we verified our proposed method with the example of reconstructing the traffic flow data from 6:00 a.m. on the Tuesday of the fourth week of August 2020.

### 3.3. Detailed Network Parameter Setting

#### 3.3.1. Combination of Hidden Layers for Generating and Discriminating Networks

**Figure 3:**
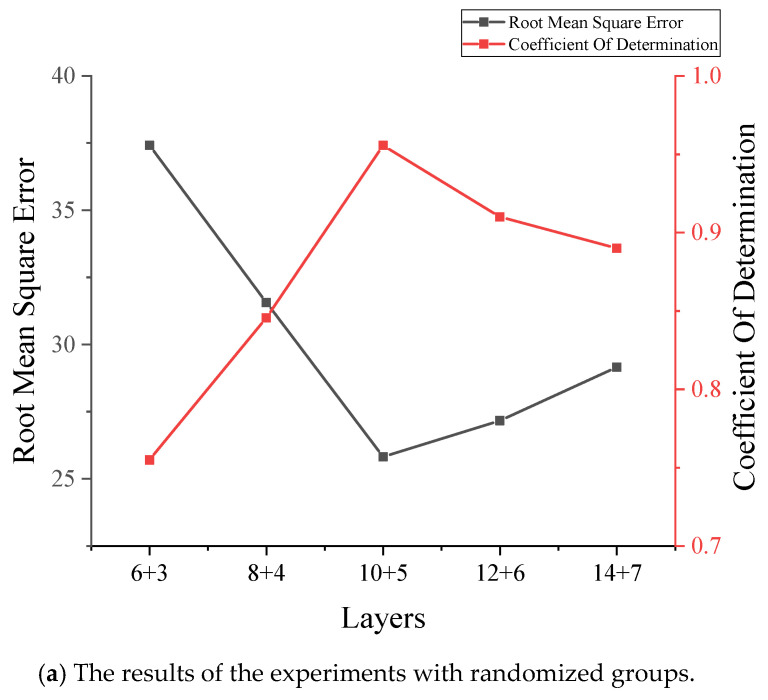

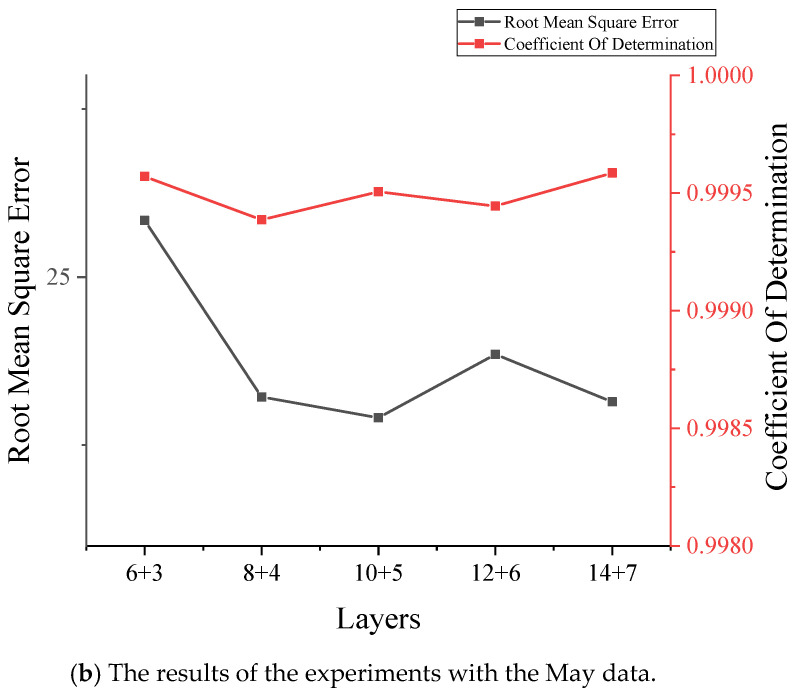
The comparison of layer combinations.

In building the network, the number of layers of the generative network should be twice that of the discriminative network. This is because the generative network contains convolutional and deconvolutional steps, while the discriminative network contains only convolutional steps. In the experimental stage, the vehicle weight data of June, July, September, November, and December were used in the dataset. Therefore, before reconstructing the signal using the proposed GAN model, this paper investigated the optimal structure using two types of data, May and a random array, and trained all models under the same conditions. The results are shown in Figure 3. As shown in Figure 3a, it can be clearly seen that the combination of 10 + 5 layers is the best structure. In Figure 3b, the $R^{2}$ values are very close, but we can still conclude that the combination of 10 + 5 is the best structure by comparing the RMSE values. The proposed GAN used a combination of 10 + 5 layers to build the network, i.e., a generator containing 10 hidden layers and a discriminator with 5 hidden layers. In the training step, both the discriminator and the generator were optimized using RMS with a learning rate of 0.0001 [45]. These hyperparameters were optimal values that were determined by the trial-and-error method after several experiments. In each training step, the discriminator was optimized five times and the generator was optimized once.

#### 3.3.2. Final Network Configuration

In order to make the GAN better adapted to the engineering status of the Jiangdong Bridge, the structure of the data reconstruction network underwent some minor changes. The convolutional kernel, step, and the sizes were slightly adjusted to match the input and output data size. In contrast, the whole network architecture, the type of layers, and the activation function remained unchanged. We were inspired by the network proposed by Lei et al. for the purpose of reducing training parameters and improving efficiency [38]. Therefore, the BN layer was added to the first layer of the generative network, as well as to the first and last layers of the discriminative network, based on the original network. As shown in Table 1, the second-dimensional design sizes of the input and output sizes of the generator were 9 and 3, which were consistent with the existing and missing data. Table 1 and Table 2 show the detailed structure of the generator (containing 10 hidden layers) and discriminator (containing 5 hidden layers). This distinguished whether the input data of size 1536 × 3 × 1 were actual data or not, and the model errors were backpropagated to update the hyperparameters in the neural network. These models were built and implemented on TensorFlow (GPU), an open-source machine learning library in python 3.7 [46]. The computer used to train this network was composed of a GTX 960 graphics card, an i78700K CPU, and 16 GB of RAM.

### 3.4. Experimental Steps

To reconstruct the traffic flow data from 6:00 a.m. on the Tuesday of the fourth week of August 2020, this paper selected the traffic flow data from 6:00 a.m. on the Tuesday of each week in June, July, September, November, and December 2020 to use for training the neural network. After the training was completed, it was used to reconstruct the traffic flow data from 6:00 a.m. on the Tuesday of the fourth week of August. It is worth noting that the October traffic data were not used in the experiment because people travel significantly more during the Chinese National Day holiday in October.

### 3.5. Data Pre-Processing

Before the formal calculation, the data collected by the WIM system needed to be preprocessed. The form of data processing was as follows:

(1) The collected data were sliced and processed, and each day’s data were divided into 24 parts, with each part containing one hour of data;
(2) The time period that was missing was selected, along with the traffic data for that time period for the remaining three weeks of the month to which the time period belonged;
(3) The selected three weeks of traffic data were combined into a matrix X.

After the statistics found that the hourly traffic flow was not greater than 1536, a matrix of 1536 × 9 was constructed. It is worth noting that more than 90% of the data collected were from small cars with only two axles. Therefore, the first and second axle weights were selected as part of the dataset. In addition, considering the damage that large heavy vehicles with more than two axles can cause to bridges, the total weight data were also used to represent large heavy vehicles in this paper. In the X matrix, columns 1, 2, and 3 were the total vehicle weight, first axle weight, and second axle weight of the selected first week, respectively. Columns 4, 5, and 6 were the total vehicle weight, first axle weight, and second axle weight of the selected second week, respectively. Columns 7, 8, and 9 were the total vehicle weight, first axle weight, and second axle weight of the selected third week, respectively. If there were less than 1536 datasets for a particular week, then the X matrix was filled with zeros to the size of 1536 × 9. The X matrix was used as the input training set for the generator, and the reconstructed data of the Y matrix of size 1536 × 3 was the output. The Y matrix was the requested missing dataset.

**Figure 4:**
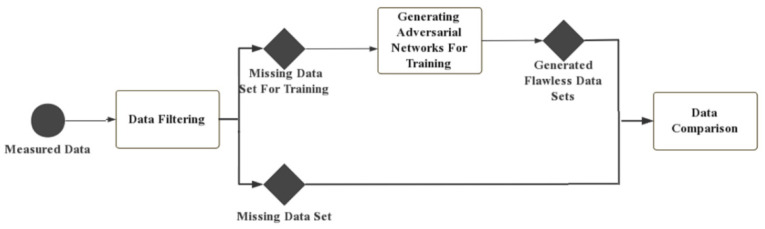
The experimental procedure.

In the experimental phase, the month with no missing data was selected as the experimental subject. As mentioned above, the data for a random three weeks of each month for a certain period were selected as the input for the generator and the data for that period for the remaining week was used as the input for the discriminator. Since the purpose of the adversarial discriminator is to distinguish the authenticity of the output data of the generator, the size of the data matrix of the input discriminator was 1536 × 3, representing the total weight, first axle weight, and second axle weight of the vehicles passing the automatic weighing system during that period. The output size of the last layer was designed to be 1 × 1 × 1, and this was the result of the discriminator’s determination. The experimental process is shown in Figure 4.

### 3.6. Selection of Number of Training

**Figure 5:**
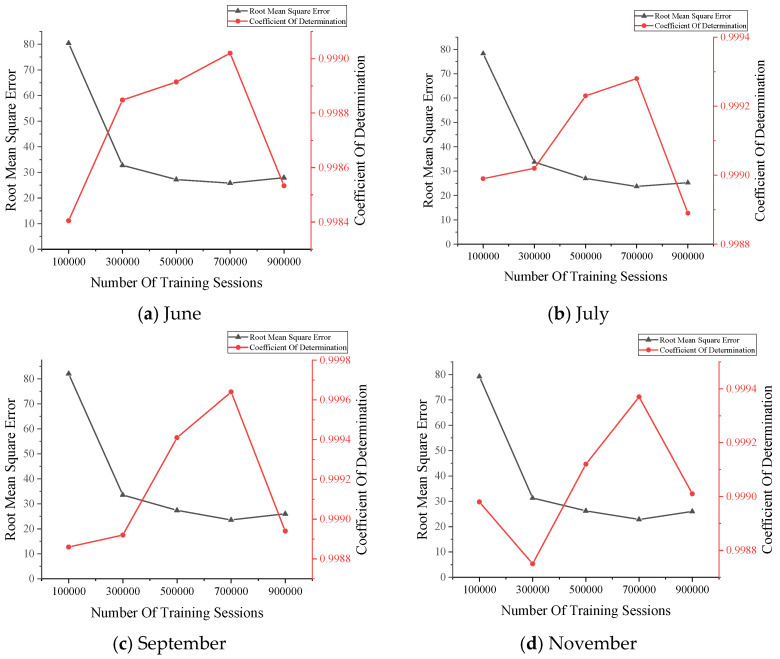

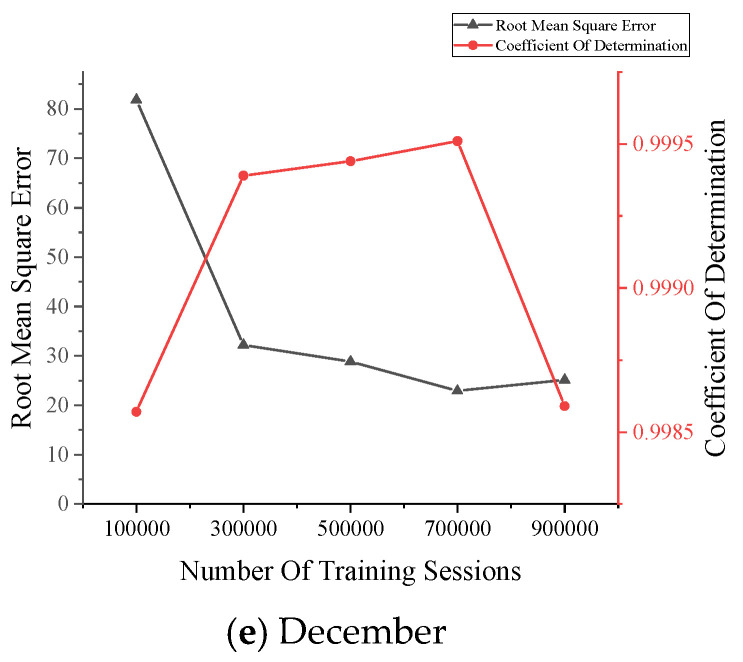
The data reconstruction results.

In this structure, the number of training counts is a crucial parameter to reconstruct the data correctly. Five different training counts were compared in this paper. Five training times of 100,000, 300,000, 500,000, 700,000, and 900,000 times were performed on the data from 6 a.m. on every Tuesday in June, July, September, November, and December of 2020, respectively. The RMSE values and $R^{2}$ values were calculated separately for the obtained reconstructed dataset, and the results are shown in Figure 5.

The above reconstruction results show that, according to the principle of ensuring that the RMSE value is small enough while guaranteeing a larger $R^{2}$ value, the more appropriate number of training times is 700,000 times.

### 3.7. Data Reconstruction Results

**Figure 6:**
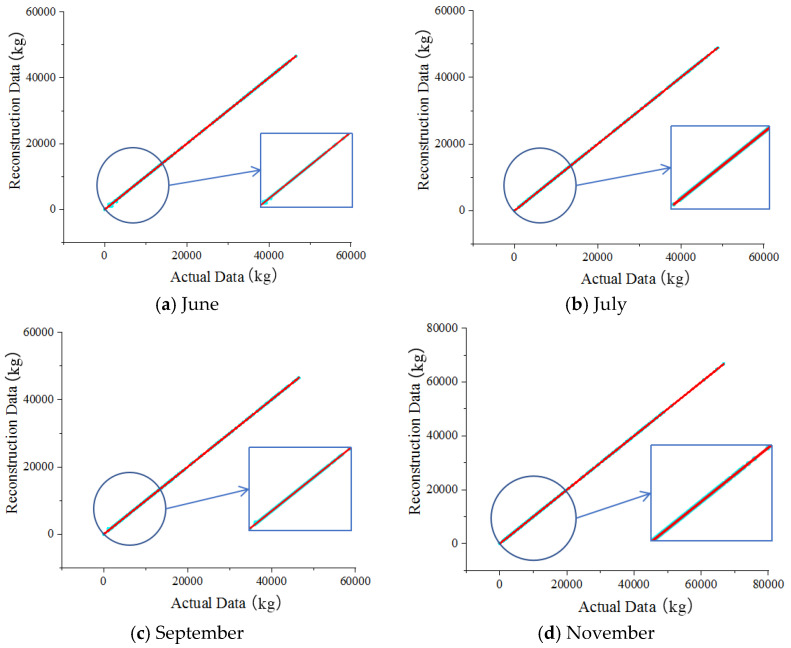

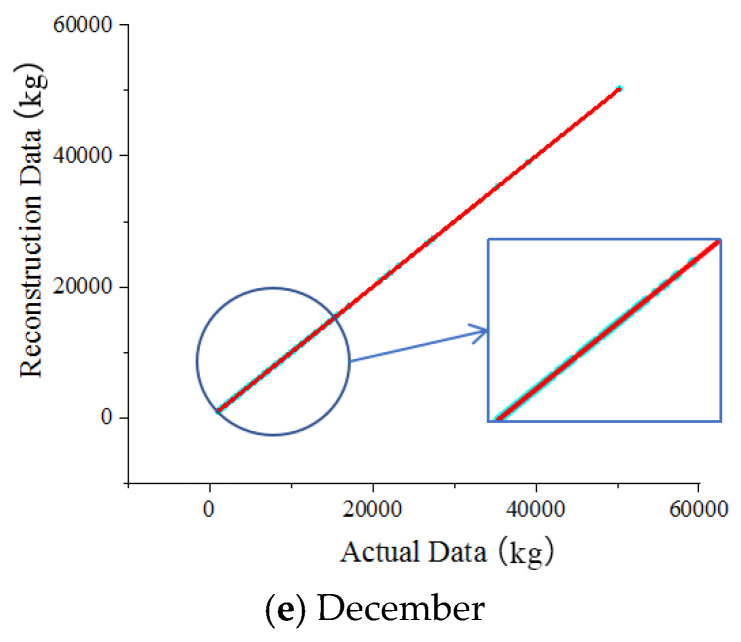
The comparison of the gross vehicle weight data.

Based on the training results, the scatter plots of the traffic flow data that were generated by the generator of the GAN after 700,000 training sessions for June, July, September, November, and December 2020 were plotted, as shown in Figure 6.

**Figure 7:**
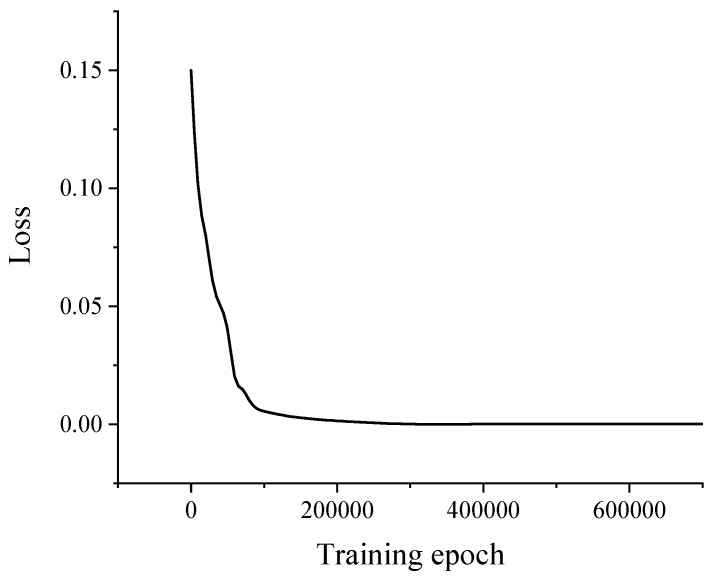
The training error for the generator network.

In Figure 6, the horizontal and vertical coordinates represent the actual data and the reconstructed data, respectively, and the scatter points in the figure are uniformly distributed over the image of y = x for two weeks, which demonstrates that the proposed model reconstructed the gross vehicle weight data adequately. Furthermore, there is a good agreement between the actual data and the reconstructed data. Thus, the proposed GAN could accurately capture and reconstruct the trained dataset’s potential features and distribution patterns. Figure 7 shows the results of the training loss of the generator network.

To better represent the usability of the GAN, this paper used the reconstruction methods described in the introduction to reconstruct the traffic flow data from the Hangzhou Jiangdong Bridge and compared the RMSE values of the datasets that were generated by the GAN model using these methods. These methods were selected because they can be used for the reconstruction of time series data, which is similar to the goal of the completion of the time series dataset for the bridge WIM system in this paper. The results are presented in Table 3.

Due to the lack of uniform parameters among the different methods, the traditional RMSE comparison was still used in this paper. Table 3 shows that the GAN had a better performance in generating the missing traffic flow data by comparing the RMSE values of the dataset that was generated by the GAN with those generated by other methods.

## 4. Conclusions

In this study, a new deep convolution-based GAN method was proposed to reconstruct missing data in the case of data transmission failure or sensor failure in automatic bridge weighing systems. The whole deep learning method, including data preprocessing, network structure, and loss function, was introduced. Then, the dataset reconstruction was performed by inputting the processed missing dataset into the GAN. Finally, the reconstructed data were compared to the actual data. The computational results were validated using the engineering example of the Hangzhou Jiangdong Bridge to verify the method’s effectiveness. The main conclusions of the study are as follows:

1. The traffic flow data that were collected by the automatic weighing system of the Hangzhou Jiangdong Bridge were selected for the experiments. Then, the training of the generating network G and the discriminating network D was carried out. After the experiments, the generated dataset that was obtained by the generator was found to be the closest to the actual dataset when the training number was 700,000 times.
2. The traffic flow data that were collected by the automatic weighing system of the Hangzhou Jiangdong Bridge were selected for the experiments. The training of the generating network G and the discriminating network D was carried out. The experiments showed that when the combination of network layers was 10 + 5, the generated dataset that was obtained by the generator was the closest to the actual dataset.
3. The GAN model that was proposed in this paper could reconstruct the field-measured vehicle weight and axle weight data well. The decomposition of the reconstructed dataset helped load identification and safety assessment. Using the data from the automatic weighing system installed on the Hangzhou Jiangdong Bridge, the data for June, July, September, November, and December were tested. The results were compared to the actual values. The results verified the applicability of the proposed GAN model in practical engineering. The proposed GAN model could accurately capture and reconstruct the overall features and specific details of the actual dataset.

## Figures and Tables

**Table 1:** The hidden layers of the generator.

Layers	Input Size	Output Size	Number of Convolution Kernels	Convolution Kernel Size	Convolution Step	Activation
Conv + BN	1536 × 9	128 × 7	32	12 × 3	12 × 1	leaky_relu
Conv + BN	128 × 7	32 × 5	64	4 × 3	4 × 1	leaky_relu
Conv + BN	32 × 5	8 × 3	128	4 × 3	4 × 1	leaky_relu
Conv + BN	8 × 3	3 × 2	256	4 × 2	2 × 1	leaky_relu
Conv + BN	3 × 2	1 × 1	512	3 × 2	1 × 1	leaky_relu
UConv + BN	1 × 1	4 × 1	256	4 × 1	1 × 1	relu
UConv + BN	4 × 1	8 × 1	128	2 × 1	2 × 1	relu
UConv + BN	8 × 1	32 × 1	64	4 × 1	4 × 1	relu
UConv + BN	32 × 1	128 × 2	32	4 × 2	4 × 1	relu
UConv	128 × 2	1536 × 3	1	12 × 2	12 × 1	tanh

**Table 2:** The hidden layers of the discriminator.

Layers	Input Size	Output Size	Number of Convolution Kernels	Convolution Kernel Size	Convolution Step	Activation
Conv	1536 × 3	128 × 2	32	12 × 2	12 × 1	leaky_relu
Conv + BN	128 × 2	32 × 1	64	4 × 2	4 × 1	leaky_relu
Conv + BN	32 × 1	8 × 1	128	4 × 1	4 × 1	leaky_relu
Conv + BN	8 × 1	3 × 1	256	4 × 1	2 × 1	leaky_relu
Conv + BN	3 × 1	1 × 1	512	3 × 1	1 × 1	sigmoid

**Table 3:** The comparison of the RMSE values of several methods.

Method	June	July	September	November	December
GAN	25.8	23.7	23.6	22.8	22.9
TRMF	40.0	38.2	27.2	29.1	32.3
MissForest	37.9	43.2	27.9	31.7	28.7
SVD	35.6	40.0	31.5	28.6	35.0
Multiple Interpolation	51.8	47.1	44.2	45.3	41.1

## Data Availability

The data that support the findings of this study are available from the corresponding author upon reasonable request.
